# Validation limitations and reporting gaps in a machine learning model for mandibular third molar impaction prediction

**DOI:** 10.1016/j.jobcr.2026.101441

**Published:** 2026-03-20

**Authors:** Carlos Fernando Mourão, Luiz Eduardo Juliasse, Rodrigo dos Santos Pereira

**Affiliations:** a Department of Basic and Clinical Translational Sciences, Tufts University School of Dental Medicine, Boston, MA, USA; b Department of Clinical Dentistry, Universidade Federal do Rio de Janeiro, Rio de Janeiro, Brazil

Dear Editor,

We read with interest the article by Jidiya et al.,^1^ which presents a machine learning model for predicting mandibular third molar impaction using demographic and radiographic variables. While applying XGBoost with SHAP-based interpretability addresses a highly relevant clinical challenge, specific methodological concerns warrant discussion before broader clinical implementation.

First, the validation framework relies solely on internal validation via a 70:30 random split from a single institution.^1^ This approach inherently yields optimistic performance estimates. Temporal or geographic validation using independent datasets is essential to demonstrate true generalizability, as recommended by established prediction model guidelines.^2^, ^3^, ^4^.

Second, complication-specific predictions should be interpreted with high caution. Events such as cyst formation (n = 12, 8%) and nerve involvement (n = 8, 5%) represent extremely low event counts within the dataset of 148 impacted teeth.^1^ These frequencies are insufficient to support stable model discrimination and significantly increase the risk of overfitting. Such findings should be considered strictly exploratory until replicated in larger cohorts.^3^

Third, key reporting elements are absent. Although two oral radiologists independently evaluated all radiographs, no inter-rater reliability metric is reported for individual feature classifications.^1^ Quantifying observer agreement is necessary to assess measurement bias in key predictors, aligning with TRIPOD + AI standards.^4^ Furthermore, neither the trained model weights nor the analytical code are publicly available, limiting reproducibility and independent evaluation.^4^

We commend the authors for this proof-of-concept study and encourage multi-institutional external validation, cautious interpretation of rare-event predictions, and strict alignment with AI reporting standards.^2^, ^3^, ^4^.

## Patient's/guardian's consent

Not applicable;

## Informed consent statement

Not applicable. This Letter to the Editor does not involve any human participants or patient data.

## Ethical clearance

Not required.

## Ethical approval statement

This manuscript is a Letter to the Editor providing commentary on a previously published article. No original research involving human subjects, animals, or patient data was conducted for this work. Therefore, ethical approval was not required.

## Sources of funding

This research did not receive any specific grant from funding agencies in the public, commercial, or not-for-profit sectors.

## Declaration of competing interest

The authors declare that they have no known competing financial interests or personal relationships that could have appeared to influence the work reported in this paper.
